# New record of nuclear DNA amounts of some Zingiberaceae species from North east India

**DOI:** 10.1016/j.dib.2017.12.063

**Published:** 2018-01-03

**Authors:** Supriyo Basak, Latha Rangan

**Affiliations:** a Department of Biosciences and Bioengineering, Indian Institute of Technology Guwahati, Assam, 781039, India; b Key Laboratory for Plant Diversity and Biogeography of East Asia, Kunming Institute of Botany, Chinese Academy of Sciences, Kunming 650201, China

## Abstract

Members of the family Zingiberaceae are important medicinal plants and have great economic significance. Some taxonomic issues are still pending within the family and the genome size estimates of many species are still very scarce. Therefore, studies concerning genome size can provide complementary data that may be useful to characterize the family on whole. Genome size estimate have been used to characterize four Northeast Indian taxa of the family Zingiberaceae occurring in the wild in addition to that of a sacred cultivated species. In this data article we have provided genome size estimates of four species based on flow cytometry for the first time. This data will be valuable for genomic and molecular authentication of these species for all future studies.

**Specifications table**

**Table:** 

Subject area	Applied Biodiversity
More specific subject area	Evolutionary genomics
Type of data	Histogram, Tables (genome size)
How data was acquired	Fluorescence based relative detection of propidium iodide bound to nucleus.
Data format	Processed
Experimental factors	Experiment was conducted in room temperature. The data was based on three days repeated measurements on three different individuals
Experimental features	Co-processing of nuclei released from a standard and a test species
Data source location	Guwahati, Assam, India
Data accessibility	All data presented in this article

**Value of data**

• Data for the first time show the nuclear DNA content of *Globba bulbifera, Boesenbergia longiflora, Zingiber* sp (Moran) and *Alpinia nigra.*
• These data will be helpful in species delineation.
• Data will help investigators in planning genetic diversity studies, breeding and sequencing.
• Relevant for researchers investigating the phenology of these plants for climate change.

## 1 Data

**Fig. 1:**
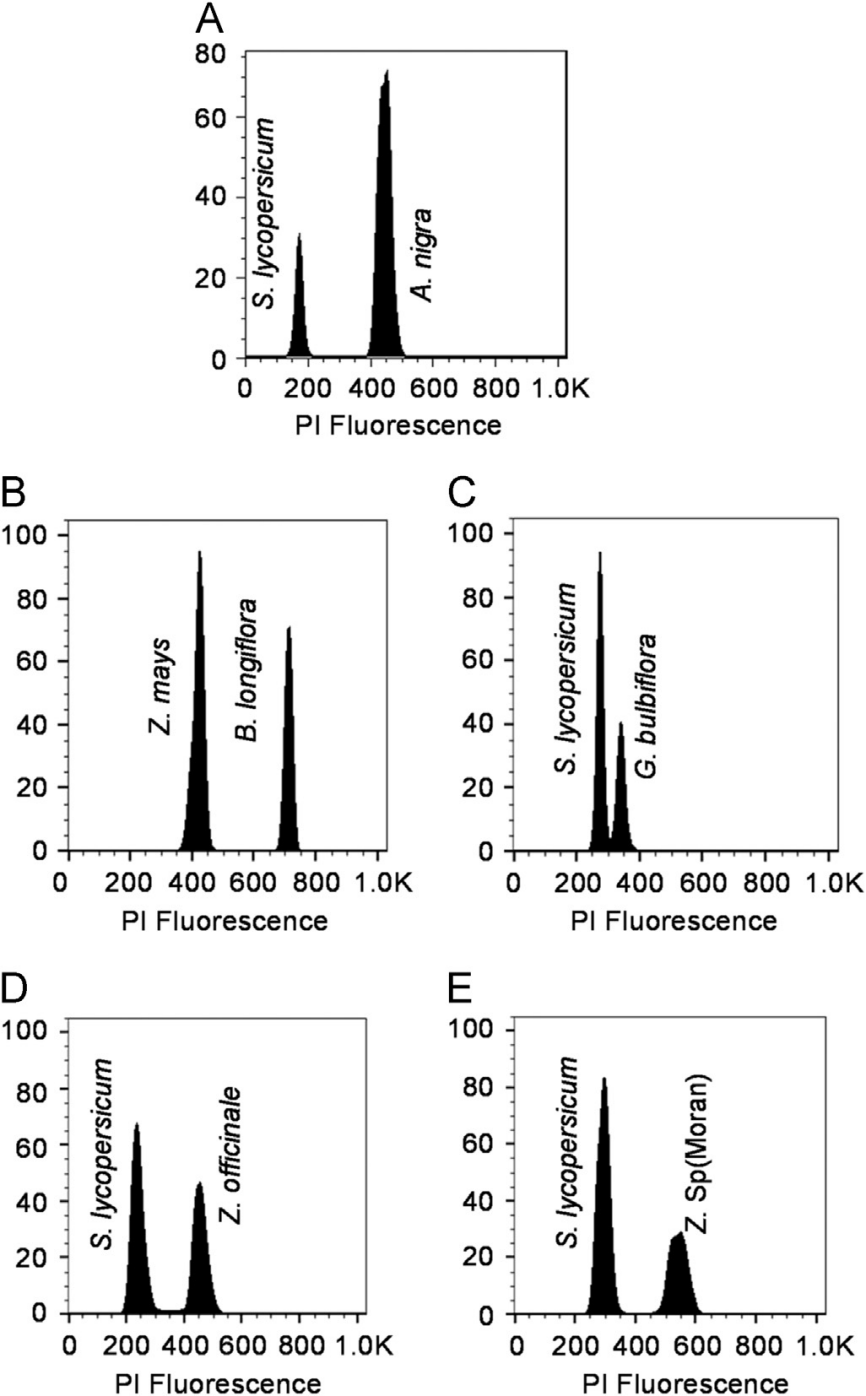
Histogram showed relative fluorescent intensity for Zingiberaceae species using internal standards. The species in the panel are: (A) *A. nigra,* (B) *B. longiflora,* (C) *G. bulbifera,* (D) *Z. officinale* (E) *Z.* sp (Moran). Reference standards used were *Solanum lycopersicum* ‘Stupicke’ (2C = 1.96 pg), *Zea mays* ‘CE-777’ (2C= 5.43 pg).

The origin of plant material and subsequent previous literature regarding the species is listed in Table 1. The flow cytometric histograms of the five species are shown in Fig. 1. As shown in the Table 2 and Fig. 1 the coefficient of variation of the fluorescence peak is less than 5% depicting its good quality for nuclear DNA content estimation.

**Table 1:** Description of the Zingiberaceae plants under consideration with their literature report on previous genome size investigation.

Serial no	Species name	Voucher No	Literature reports on nuclear DNA content
1	*Alpinia nigra* (Gaertn.) B.L.Burtt	10346	No report
2	*Boesenbergia longiflora* (Wallich) Kuntze	10343	No report
3	*Globba bulbifera* Roxb.	10342	No report
			
4	*Zingiber officinale* Rosc.	10344	12.05 ( 4C [3])
			3·60 (2C [4])
5	*Zingiber* sp (Moran)	10345	No report

**Table 2:** Nuclear DNA content reported in this investigation.

Serial No	Species Name	Genome size (2C, pg)	CV	1C (pg)	1C (Mbp)	Standard used
1	*A. nigra*	4.58 ± 0.02	2.50	2.29	2239	S
2	*B. longiflora*	9.02 ± 0.05	3.46	4.51	4410	Z
3	*G. bulbifera*	2.53 ± 0.01	3.83	1.26	1237	S
4	*Z. officinale*	3.61 ± 0.09	4.95	1.80	1765	S
5	*Z.* sp (Moran)	3.68 ± 0.02	1.97	1.84	1799	S

S: *Solanum lycopersicum;* Z: *Zea mays*

## 2 Experimental design, materials and methods

### 2.1 Plant material

Out of the five Zingiberaceae species, rhizomes of four plants were collected from their natural habitat. Rhizome of Z. sp (moran) was collected from the local market and grown in the green house under controlled condition. The herbarium voucher of the studies material was deposited in the Botanical Garden Herberium of Department of Botany, Gauhati University, Assam India (Accredited by New York, USA).

### 2.2 Standards for flow cytometric estimation of nuclear DNA content

Standard plants used for estimation of nuclear DNA content were *Solanum lycopersicum* cv. Stupicke (2C=1.96 pg) and *Zea mays* CE-777 (2C=5.43 pg) received on request. These standards were grown in the green house along with the study material in the similar fashion.

### 2.3 Isolation and staining of nuclei

Nuclei were released from the intact plant tissue by chopping with a double edged sterile razor blade in presence of 1 ml of propidium iodidle/hypotonic citrate buffer. Standard and the test plants were chopped together and filtered with a 30 µm nylon mesh. Propidium iodide (PI) (25 mg/ml) and RNaseA (2 mg/ml) were added to the filtered suspension and incubated for 10 min in dark [1].

### 2.4 Measurement of nuclear DNA content and data analysis

Nuclear DNA content was measured in a FACS Calibur flow cytometer (BD Bioscience, USA). The experimental set up was as described previously [1]. In brief, in a fixed voltage and gain settings, the nuclei were captured in three dot plot and one histogram plot. The three dot plots were forward scatter (FSC) vs side scatter (SSC), SSC vs FL2A and FL2A vs FL2W. The size and granularity of the captured nuclei was shown by FSC vs SSC plot. The position of the PI nuclei was obtained in the SSC vs FL2 plot. The singlet nuclei was segregated from the clumped nuclei in the FL2A vs FL2W plot by selecting proper gating within this dot plot. The number of PI stained nuclei of the test and standard species was recorded in the Frequency vs Fl2A histogram plot. For each species, atleast 10,000 nuclei were recorded at a flow rate of 20–50 nuclei per second. The product of the nuclear DNA content (2 C, in pg) of standard species and ratio of the mean fluorescent intensity of test and standard species give the estimate of the nuclear DNA content of the test species. The raw data was processed using FlowJo v.7.6.5 (FlowJo, Tree Star Inc, Ashland, OR). The step-by-step gating procedures in FlowJo were according to the methodology described as previously [1]. The nuclear DNA content (2 C, pg) was converted to base pairs by considering 1 pg of DNA corresponds to 978 Mbp [2].
